# The relationship between sleep quality and daytime dysfunction among college students in China during COVID-19: a cross-sectional study

**DOI:** 10.3389/fpubh.2023.1253834

**Published:** 2023-11-10

**Authors:** Wei Ji, Liyong Shi, Xinjun Lin, Zhiyong Shen, Qingquan Chen, Duanhong Song, Pengxiang Huang, Zhihuang Zhao, Jimin Fan, Yiming Hu, Mianmian Xie, Jiaohong Yang, Xiaoyang Chen

**Affiliations:** ^1^The Second Clinical College of Fujian Medical University, Quanzhou, Fujian Province, China; ^2^Department of Respiratory and Critical Care Medicine, The Second Affiliated Hospital of Fujian Medical University, Quanzhou, Fujian Province, China; ^3^Department of Respiratory and Critical Care Medicine, Jinjiang City Hospital, Quanzhou, Fujian Province, China; ^4^The School of Public Health, Fujian Medical University, Fuzhou, Fujian Province, China; ^5^National Center for Chronic and Noncommunicable Disease Control and Prevention, Chinese Center for Disease Control and Prevention, Beijing, China

**Keywords:** sleep quality, daytime dysfunction, PSQI, college students, COVID-19

## Abstract

**Objective:**

College Students’ sleep quality and daytime dysfunction have become worse since the COVID-19 outbreak, the purpose of this study was to explore the relationship between sleep quality and daytime dysfunction among college students during the COVID-19 (Corona Virus Disease 2019) period.

**Methods:**

This research adopts the form of cluster random sampling of online questionnaires. From April 5 to 16 in 2022, questionnaires are distributed to college students in various universities in Fujian Province, China and the general information questionnaire and PSQI scale are used for investigation. SPSS26.0 was used to conduct an independent sample t-test and variance analysis on the data, multi-factorial analysis was performed using logistic regression analysis. The main outcome variables are the score of subjective sleep quality and daytime dysfunction.

**Results:**

During the COVID-19 period, the average PSQI score of the tested college students was 6.17 ± 3.263, and the sleep disorder rate was 29.6%, the daytime dysfunction rate was 85%. Being female, study liberal art/science/ engineering, irritable (due to limited outdoor), prolong electronic entertainment time were associated with low sleep quality (*p* < 0.001), and the occurrence of daytime dysfunction was higher than other groups (*p* < 0.001). Logistics regression analysis showed that sleep quality and daytime dysfunction were associated with gender, profession, irritable (due to limited outdoor), and prolonged electronic entertainment time (*p* < 0.001).

**Conclusion:**

During the COVID-19 epidemic, the sleep quality of college students was affected, and different degrees of daytime dysfunction have appeared, both are in worse condition than before the COVID-19 outbreak. Sleep quality may was inversely associated with daytime dysfunction.

## 1. Introduction

One-third of a person’s life is spent in sleep. The quality of sleep often determines the quality of personal life (1, 2). With the accelerating pace of life in contemporary society, different degrees of sleep problems and daytime dysfunction have emerged. Before the outbreak of the COVID-19 epidemic, more than 200 million people in China had varying degrees of sleep quality problems and daytime dysfunction (1). Since the outbreak of the COVID-19 epidemic, according to the Chinese People’s Sleep Quality Report (2020), about nearly 300 million people in China have different degrees of sleep quality problems and daytime dysfunction, and nearly two-thirds of them claim that they suffer from insomnia and other symptoms (2, 3). College students who are in the late stage of adolescence and about to enter society are sensitive to changes in the external environment. Sleep problems and daytime dysfunction of this group have become more severe under the influence of changes in study and life patterns, and epidemic prevention policies in the COVID-19 era (4, 5). Recent studies have showed that 36.1–50.1% of college students had different degrees of sleep quality problems and daytime dysfunction (6). In 2022, due to the recurrence of the COVID-19 epidemic, colleges and universities insisted on the general strategy of “external prevention of importation and internal prevention of rebound” (Specific actions include tightening controls on the student movement in COVID-19 epidemic regions and boosting isolation and supervision of people accessing the campus, ensuring that students do not leave campus unless required, and so on. Before the COVID-19 outbreak, these measures had never been implemented.), scientifically formulated the spring back-to-school program, and strengthened the health management of teachers, students, and staff returning to school. Hence, the study and life patterns of college students have changed significantly. The purpose of this study is to understand and evaluate the sleep quality of college students during the COVID-19, to explore its relationship with daytime dysfunction, and to participate in investigating the sleep quality reports of students in each university during COVID-19.

## 2. Materials and methods

### 2.1. Study design

This study is a cross-sectional study with a whole random sampling form of an online questionnaire, which was distributed to college students in Fujian Province, China from April 5 to 16, 2022, and filled out by students voluntarily and anonymously. The survey has returned 6,993 questionnaires, covering 33 colleges and universities in Fujian Province. Among them, 5,379 questionnaires were valid, and the recovery efficiency was 76.9%.

### 2.2. Methodology of the study

#### 2.2.1. Sample size

The sample size was determined according to the formula (7) ( $n=\frac{\mu^{2}\alpha \pi \left(1-\pi \right)}{\delta^{2}}$ ). π is the detection rate of college students’ sleep disorders. In order to minimize the error, we set π as 0.3 [Based on the reference (6)]. δ is the allowable error, it is the maximum error of the sample rate and the overall rate that should be controlled range, we set the allowable error is 0.01π. We set α as 0.05, a confidence interval is taken as 95%, then the corresponding μ is 1.96. Using this method, we determine that n is 4,482 (people), and considering the 20% lost interview rate, 5,379 people need to be surveyed.

#### 2.2.2. Questionnaire development

**Figure 1:**
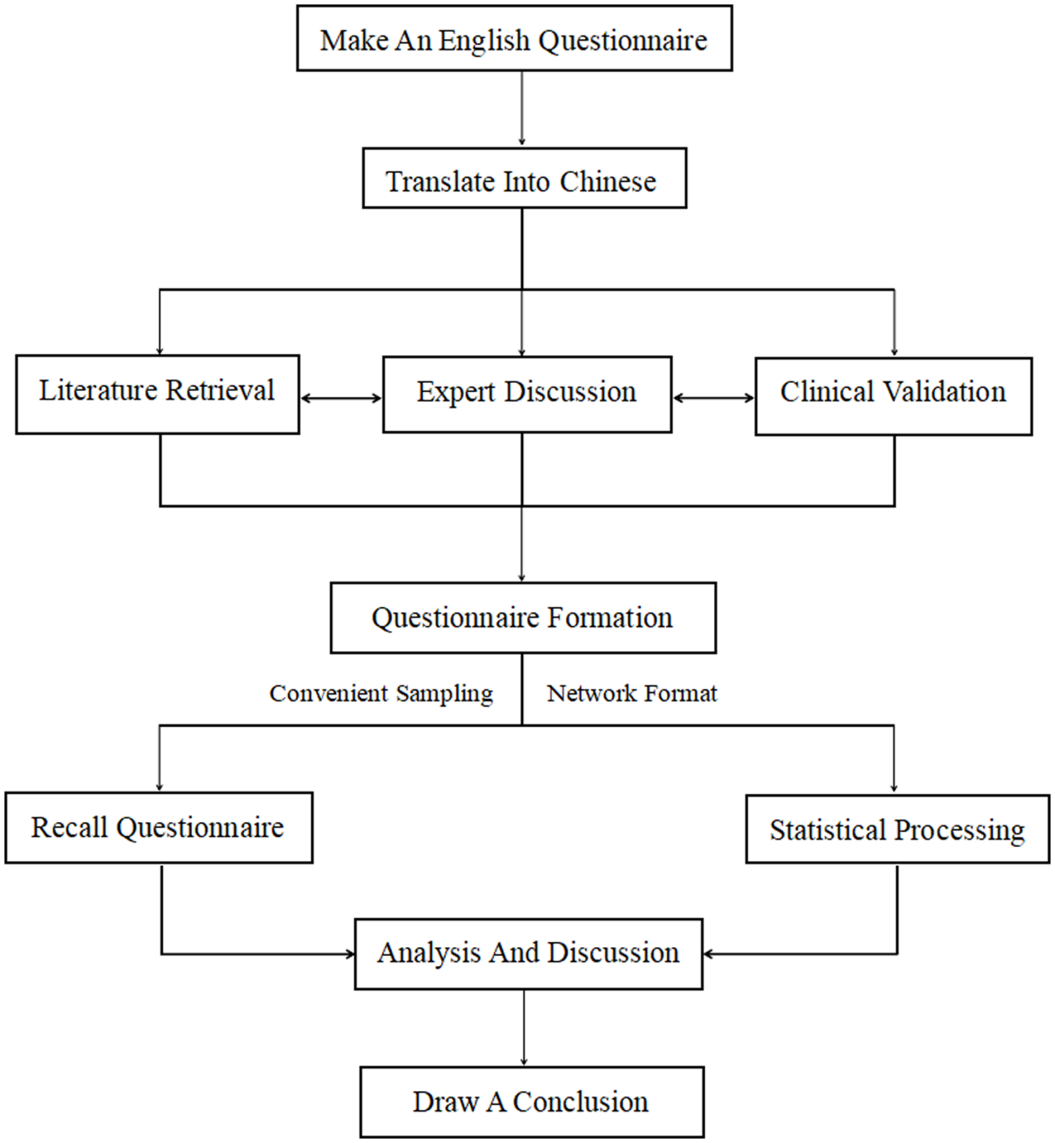
The questionnaire development process.

The questionnaire was first developed in English and then translated into Chinese, and a literature review, clinical expert discussion, and validation were conducted to check the reliability and validity related to the development of the questionnaire. The Pittsburgh Sleep Quality Index scale (8) with high reliability and validity was used, and then the online questionnaire design tool (Sleep Medicine Research Management Platform) was used to form an online questionnaire. After the questionnaire was formed, whole-group random sampling was conducted online, and the questionnaire was distributed to college students in Fujian Province, China from April 5 to 16, 2022. The study was approved by the Ethics Committee of the Second Affiliated Hospital of Fujian Medical University. Prior to completing the survey, participants were informed that participation was voluntary and that their data were anonymous. Study participants consented to their participation in the survey being published. The Questionnaire development process is shown in Figure 1.

#### 2.2.3. Questionnaire quality control

To ensure the quality of the questionnaire itself, we have conducted literature review, clinical expert discussion, and validation. To ensure the quality of respondents filling out the questionnaire, we performed a logical relationship validation of the questionnaire, such that some of the question options are mutually exclusive or inclusive. We set a certain number of questions and screened out the questionnaires with inconsistent answers to these questions. In addition, we measured the response time and screened out the questionnaires where the response time was less than 5 min. In addition, to limit duplication, each IP address and cell phone number can only fill out the questionnaire once. Finally, we conducted manual screening after the questionnaires were collected, and the questionnaires with disorderly or negative answers were invalidated.

#### 2.2.4. General information questionnaire

1. General information: It contains basic information such as age, gender, school, and major.
2. Study and life situation survey: It contains lifestyle (diet, exercise situation) and study situation (online class study status) during the COVID-19 epidemic.
3. Compliance survey: Whether you are willing to accept nighttime sleep quality monitoring? (in the form of wearable devices or smart bracelets); whether you are willing to accept follow-up?

#### 2.2.5. Pittsburgh sleep quality index

The PSQI scale was developed by scholars Buysse (8) and others and then revised by scholars Liu (9) and others. The PSQI scale was used to assess the sleep quality of subjects in the last month, and its Chinese version has good reliability with a retest reliability of 0.81, a sensitivity of 98.3%, and a specificity of 90.2% (10). The Cronbach’s α with Chinese version is 0.842 (9). After the survey, the discrete and missing data were removed according to the demographic data indicators, and the relevant items were analyzed with high internal consistency (Cronbach’s α = 0.859). The PSQI scale consists of 18 items, which is divided into 7 components. The components include sleep quality (A), time to fall asleep (B), sleep duration (C), sleep efficiency (D), sleep disturbance (E), hypnotic drug use (F), and daytime dysfunction (G). Each component is scored on a scale of 0–3, and the cumulative score of each component is the total PSQI score, which ranges from 0 to 21, with higher scores indicating poorer sleep quality. 0–5 is considered good sleep quality, 6–10 is fair sleep quality, 11–15 is poor sleep quality, and 16–21 is worse sleep quality. A total PSQI score > 7 is the criteria for determining sleep disorders (11). There is one item abnormality in the daytime dysfunction score, which is identified as a daytime dysfunction (Daytime dysfunction score ≥ 1) (12).

### 2.3. Date analysis

In this study, SPSS26.0 software was used to analyze the data. SPSS26.0 was used to conduct an independent sample t-test and variance analysis on the data, as well as to determine whether the differences among variables were statistically significant through the significant differences of each component, to test whether there were mediating effects among variables. Multi-factorial analysis was performed using logistic regression analysis. Differences were considered statistically significant at *p* < 0.05. The measurement data conforming to normal distribution were expressed as mean ± standard deviation, those not conforming to normal distribution were expressed as median (interquartile spacing), and the count data were expressed as rate or composition ratio. In addition, all statistical tests were two-sided (α = 0.05).

## 3. Results

### 3.1. General status of tested college students

**Figure 2:**
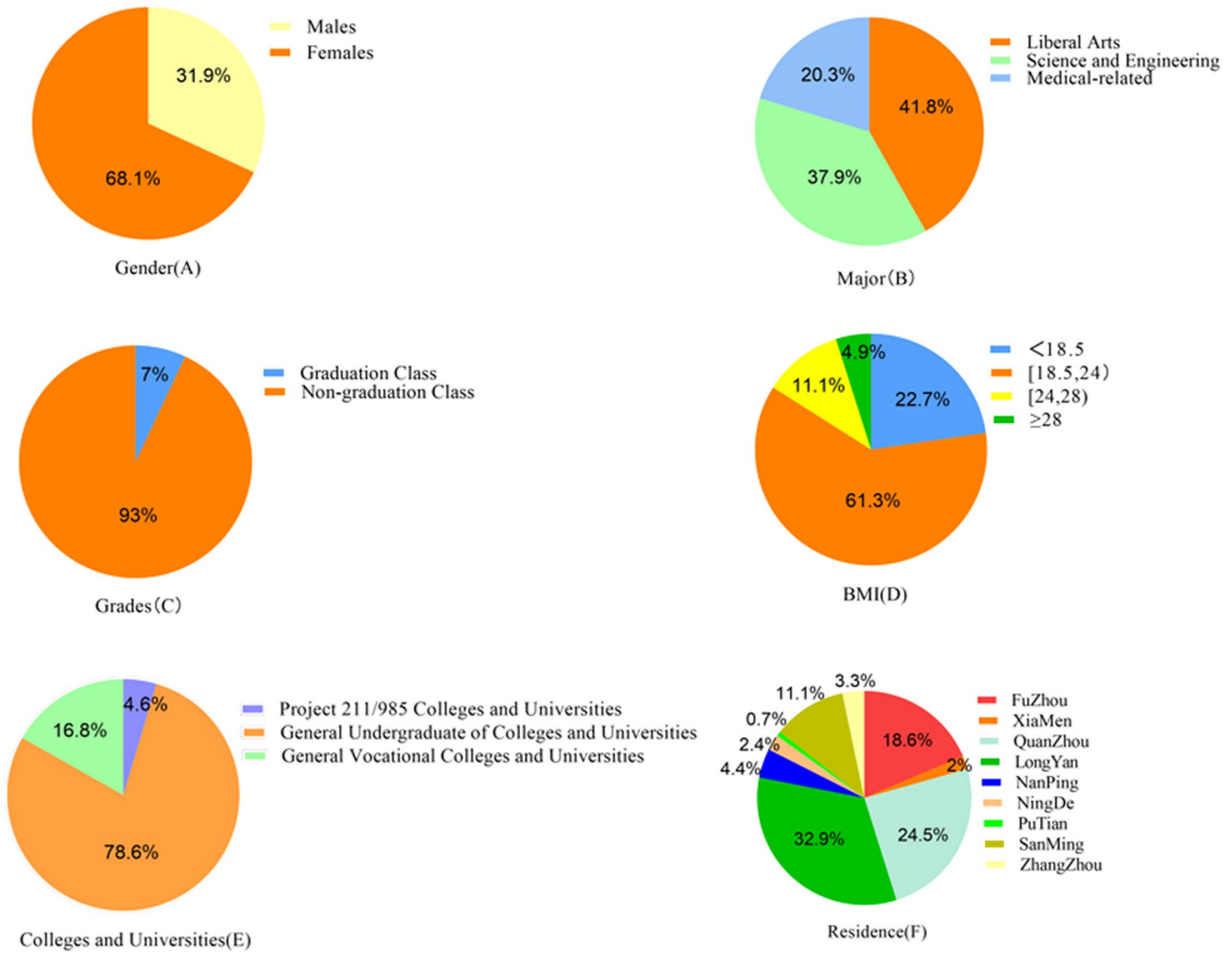
Percentage of participants with different demographic characteristics, including gender, major, grades, BMI, colleges and universities, and residence.

The age range of this sample is 17–25 years old, and the average age is “19 ± 1.4” years old. There were 1714 (31.9%) males and 3,665 (68.1%) females. There were 2,251 (41.8%) liberal arts majors, 2037 (37.9%) science and engineering majors, and 1,091 (20.3%) medical-related majors. There were 376 students (7.0%) in the graduating class and 5,003 students (93.0%) in the non-graduating class. Other demographic information is shown in Table 1 and Figure 2.

**Table 1:** General status of tested college students (*n*, %).

Variables	Categories	Participants	Constituent ratio
Gender			
	Male	1714	31.9
	Female	3,665	68.1
Major			
	Liberal Arts	2,251	41.8
	Science and Engineering	2037	37.9
	Medical-related	1,091	20.3
Grades			
	Graduation Class	376	7
	Non-graduation Class	5,003	93
BMI			
	<18.5	1,221	22.7
	[18.5,24]	3,295	61.3
	[24,28]	598	11.1
	≥28	265	4.9
Colleges and Universities			
	Project 211/985 Colleges and Universities (National Key Colleges and Universities)	246	4.6
	General Undergraduate of Colleges and Universities	4,229	78.6
	General Vocational Colleges and Universities	904	16.8
Residence			
	FuZhou	1,003	18.6
	XiaMen	109	2
	QuanZhou	1,318	24.5
	LongYan	1772	32.9
	NanPing	238	4.4
	NingDe	128	2.4
	PuTian	37	0.7
	SanMing	595	11.1
	ZhangZhou	179	3.3

### 3.2. Comparison of sleep quality and demographic differences among college students

**Figure 3:**
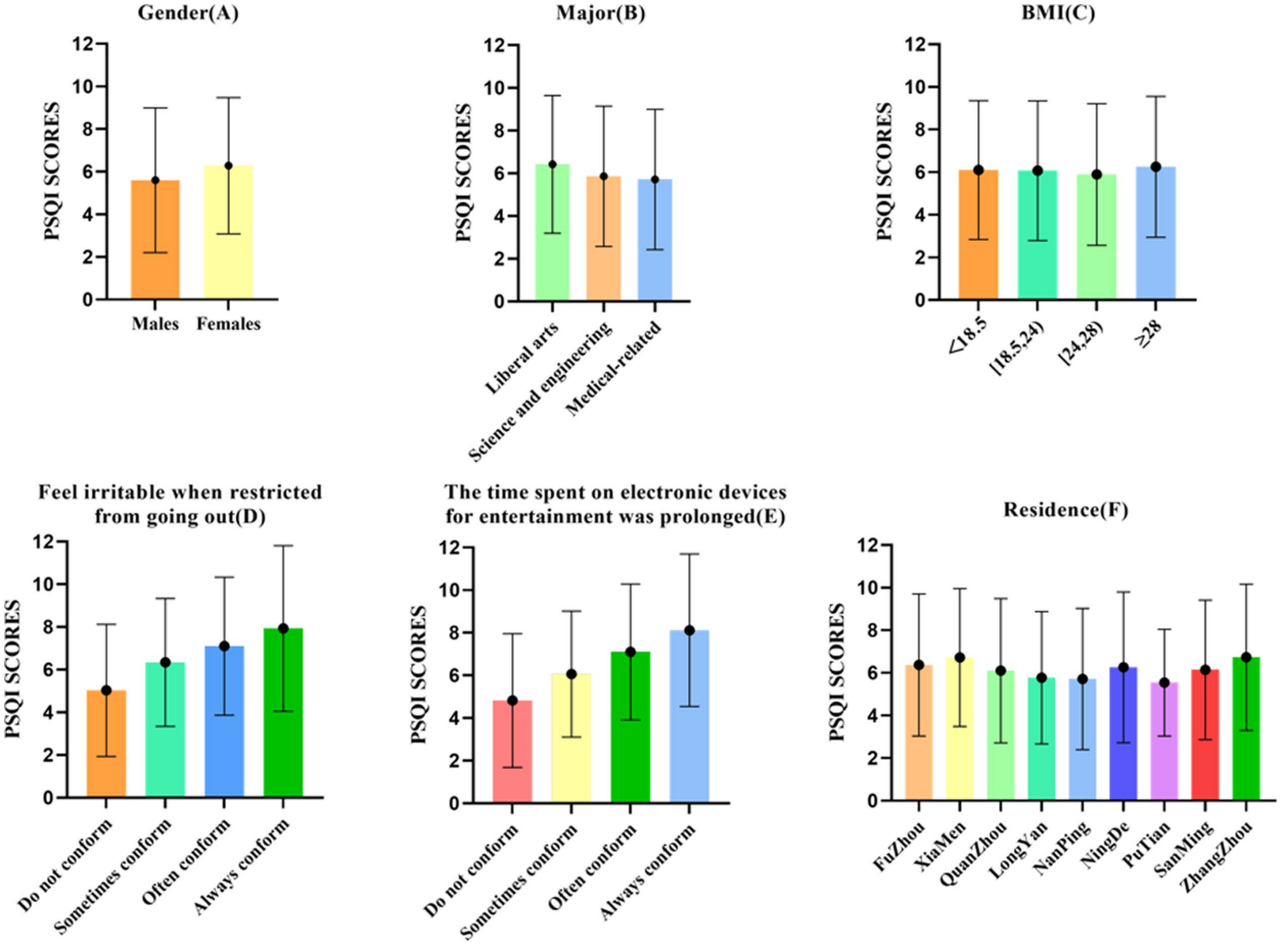
Comparison of PSQI scores, including gender, major, grades, BMI, colleges and universities, and residence.

In this study, the mean PSQI total score of college students during the COVID-19 epidemic was 6.17 ± 3.263. Differences in scores were correlated with gender, major, irritable (due to limited outdoor), and the time spent on electronic devices for entertainment was prolonged, and was not statistically significantly related to district and BMI; The differences in sleep disorder rates among college students were statistically significantly correlated with gender, major, irritable (due to limited outdoor), the time spent on electronic devices for entertainment was prolonged, and district, and was not statistically significantly related to BMI. Among 5,379 college students, the number of poor sleep quality was 1,594, the rate of sleep disorders was 29.6%, the rate of daytime dysfunction was 85% (The percentage of daytime dysfunction scores 1–2 was 31%, scores 3–4 was 33%, scores 5–6 was 21%.). Other information is summarized in Table 2 and Figure 3.

**Table 2:** Comparison of sleep quality among college students in different demographic groups (*n*, %, x̅ ± S).

Variables	Categories	The number of poor sleep quality	The rate of sleep disorders	PSQI total score	t/F	*p*	*χ^2^*	*p*
Gender					−6.944	<0.001	17.846	<0.001
	Male	442	25.8	5.6 ± 3.397				
	Female	1,152	31.4	6.28 ± 3.195				
Major					23.803	<0.001	40.868	<0.001
	Liberal arts	770	34.2	6.42 ± 3.228				
	Science and engineering	554	27.2	5.86 ± 3.286				
	Medical-related	270	24.7	5.71 ± 3.286				
BMI					0.903	0.856	0.854	0.837
	<18.5	362	29.6	6.10 ± 3.253				
	[18.5,24]	983	29.8	6.07 ± 3.273				
	[24,28]	168	28.1	5.89 ± 3.321				
	≥28	81	30.6	6.25 ± 3.303				
Feel irritable when restricted from going out					156.445	<0.001	261.945	<0.001
	Do not conform	400	19.4	5.03 ± 3.092				
	Sometimes conform	694	30.9	6.34 ± 2.994				
	Often conform	269	44.3	7.10 ± 3.230				
	Always conform	231	50.2	7.93 ± 3.870				
The time spent on electronic devices for entertainment was prolonged					192.764	<0.001	319.122	<0.001
	Do not conform	292	18	4.82 ± 3.138				
	Sometimes conform	631	27.8	6.06 ± 2.958				
	Often conform	409	40.5	7.10 ± 3.181				
	Always conform	262	55.5	8.12 ± 3.574				
Residence					4.972	0.026	48.52	<0.001
	FuZhou	343	34.2	6.37 ± 3.333				
	XiaMen	42	38.5	6.72 ± 3.238				
	QuanZhou	400	30.3	6.10 ± 3.380				
	LongYan	431	24.3	5.77 ± 3.106				
	NanPing	66	27.7	5.71 ± 3.312				
	NingDe	39	30.5	6.26 ± 3.534				
	PuTian	9	24.3	5.54 ± 2.501				
	SanMing	196	32.9	6.14 ± 3.272				
	ZhangZhou	68	38	6.73 ± 3.434				

The first value of *p* corresponds to the t/F test and the second value of *p* corresponds to the chi-square test.

### 3.3. Binary logistic regression—good and bad sleep quality

**Figure 4:**
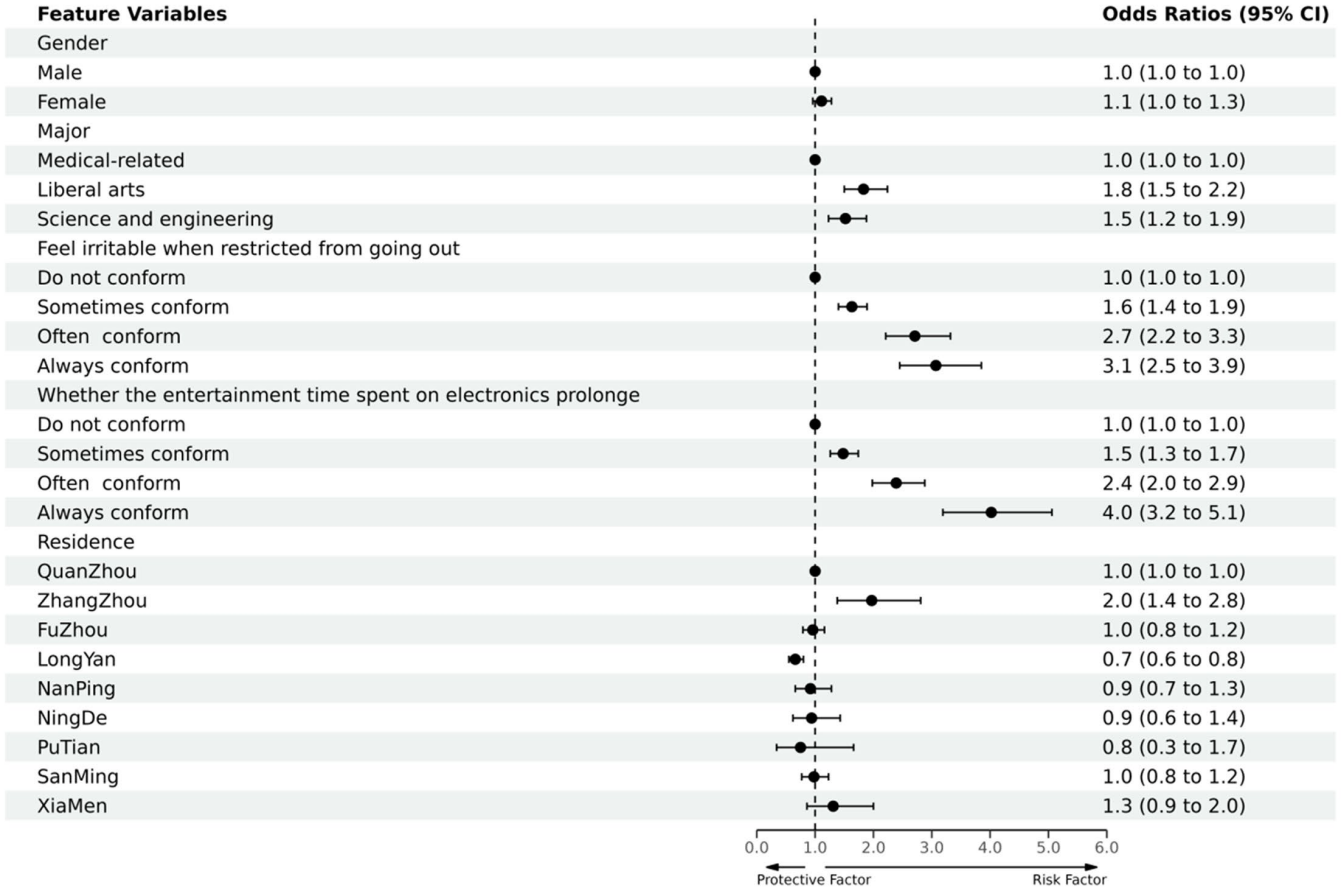
Protective and risk factors of multi-factor logistic regression analysis of college students’ sleep quality (Ending with good or bad sleep quality).

The PSQI score was used as the dependent variable (bad sleep quality = 1, good sleep quality = 0), and gender, major, irritable (due to limited outdoor), the time spent on electronic devices for entertainment was prolonged, and the district was used as independent variables in a multi-factorial unconditional logistic regression analysis (inclusion criteria were *p* < 0.05 and exclusion criteria were *p* > 0.10). The rows with β of 0 and OR of 1 in Table 3 were used as controls. The results of the analysis showed that female, liberal arts and science engineering, irritable (due to limited outdoor), the time spent on electronic devices for entertainment was prolonged, and Quanzhou city, Zhangzhou city, and Longyan city in the district were statistically significantly associated with better or worse sleep quality during the COVID-19 epidemic under the corresponding controls. This is summarized in Table 3 and Figure 4.

**Table 3:** Multi-factor logistic regression analysis of college students’ sleep quality (ending with good or bad sleep quality).

Factors	Variables	*β*	Wald *χ^2^*	OR (95%CI)	*p*
Constants		−2.179	316.6299976	0.113	<0.001
Gender					
	Male	0.000		1	
	Female	0.107	2.12	1.112(0.964–1.284)	0.145
Major					
	Liberal arts	0.607	35.595	1.834(1.503–2.239)	<0.001
	Science and engineering	0.417	14.734	1.517(1.226–1.876)	<0.001
	Medical-related	0.000	36.648	1	<0.001
Feel irritable when restricted from going out					
	Always conform	1.123	95.103	3.073(2.452–3.851)	<0.001
	Often conform	0.996	92.720	2.709(2.211–3.317)	<0.001
	Sometimes conform	0.487	41.361	1.628(1.403–1.889)	<0.001
	Do not conform	0.000	144.574	1	<0.001
The time spent on electronic devices for entertainment was prolonged					
	Always conform	1.390	138.122	4.016(3.185–5.064)	<0.001
	Often conform	0.870	83.001	2.386(1.979–2.876)	<0.001
	Sometimes conform	0.391	22.144	1.479(1.256–1.741)	<0.001
	Do not conform	0.000	172.152	1	<0.001
Residence					
	FuZhou	0.678	14.063	1.970(1.382–2.807)	<0.001
	XiaMen	−0.047	0.216	0.955(0.785–1.161)	0.642
	QuanZhou	−0.409	18.528	0.664(0.551–0.800)	<0.001
	LongYan	−0.087	0.265	0.916(0.657–1.278)	0.607
	NanPing	−0.059	0.076	0.943(0.621–1.431)	0.783
	NingDe	−0.283	0.492	0.754(0.342–1.662)	0.483
	PuTian	−0.024	0.040	0.976(0.773–1.233)	0.841
	SanMing	0.269	1.554	1.309(0.857–2.000)	0.213
	ZhangZhou	0.000	48.847	1	<0.001

^a^*β* is the regression coefficient of logistic regression analysis, which indicates how many units a dependent variable increases or decreases if the other independent variables are unchanged. ^b^Wald *χ^2^* is equal to *β* divided by its standard error squared, and is used to test whether the partial regression coefficient *β* of logistic whether is equal to 0 (The rows with *β* of 0 and OR of 1 in this table were used as controls.). OR, Odds ratio; CI, Confidence interval.

### 3.4. Ordered multi-categorical logistic regression-sleep quality(A)/daytime dysfunction(G)

The outcome variables include sleep quality (A) and daytime dysfunction (G): A/G item score was the dependent variable (very good = 0, better = 1, worse = 2, very bad = 3), and gender, major, irritable (due to limited outdoor), the time spent on electronic devices for entertainment was prolonged, and district were the independent variables in an ordered multi-categorical logistic regression (method: Fisher). The rows with β of 0 and OR of 1 in Tables 4, 5 were used as controls.

**Table 4:** Multi-factor logistic regression analysis of sleep quality among college students (Sleep quality/A).

Factors	Variables	*β*	Wald *χ^2^*	OR(95%CI)	*p*
Threshold(A)					
	Very good	0.000		1	
	Better	−0.185	4.004	0.831(0.694–0.996)	0.045
	Worse	2.303	543.647	10.003(8.243–12.140)	<0.001
	Very worse	4.618	1471.013	101.251(0.970–1.226)	<0.001
Gender					
	Male	0.000		1	
	Female	0.087	2.113	1.091(0.970–1.226)	0.146
Major					
	Liberal arts	0.403	24.166	1.497(1.274–1.758)	<0.001
	Science and engineering	0.282	10.547	1.326(1.118–1.572)	<0.001
	Medical-related	0.000		1	
Feel irritable when restricted from going out					
	Always conform	1.143	125.496	3.137(2.569–3.832)	<0.001
	Often conform	0.864	90.375	2.372(1.985–2.834)	<0.001
	Sometimes conform	0.587	92.381	1.799(1.596–2.028)	<0.001
	Do not conform	0.000		1	
The time spent on electronic devices for entertainment was prolonged					
	Always conform	1.248	143.040	3.483(2.839–4.273)	<0.001
	Often conform	1.042	166.867	2.835(2.420–3.320)	<0.001
	Sometimes conform	0.620	91.447	1.859(1.637–2.111)	<0.001
	Do not conform	0.000		1	
Residence					
	FuZhou	0.173	1.224	1.189(0.875–1.614)	0.269
	XiaMen	−0.194	5.207	0.823(0.697–0.973)	0.022
	QuanZhou	−0.268	11.647	0.765(0.656–0.892)	0.001
	LongYan	−0.468	10.936	0.626(0.474–0.826)	0.001
	NanPing	0.097	0.294	1.101(0.777–1.562)	0.588
	NingDe	−0.389	1.461	0.678(0.361–1.274)	0.227
	PuTian	−0.398	15.130	0.672(0.550–0.821)	0.000
	SanMing	−0.080	0.176	0.923(0.634–1.343)	0.675
	ZhangZhou	0.000		1	

^a^*β* is the regression coefficient of logistic regression analysis, which indicates how many units a dependent variable increases or decreases if the other independent variables are unchanged. ^b^Wald *χ^2^* is equal to *β* divided by its standard error squared, and is used to test whether the partial regression coefficient *β* of logistic whether is equal to 0 (The rows with *β* of 0 and OR of 1 in Table 4 was used as controls.). OR, Odds ratio; CI, Confidence interval.

**Table 5:** Multi-factor logistic regression analysis of sleep quality among college students (Daytime dysfunction/G).

Factors	Variables	*β*	Wald *χ^2^*	OR(95%CI)	*p*
Threshold(G)					
	Very good	0.000		1	
	Better	0.723	63.658	2.060(1.725–2.461)	<0.001
	Worse	2.255	556.638	9.539(7.909–11.505)	<0.001
	Very worse	4.070	1484.867	58.584(47.628–72.060)	<0.001
Gender					
	Male	0.000		1	
	Female	0.270	22.015	1.311(1.171–1.467)	<0.001
Major					
	Liberal arts	0.413	27.209	1.511(1.294–1.765)	<0.001
	Science and engineering	0.349	22.205	1.483(1.258–1.748)	<0.001
	Medical-related	0.000		1	
Feel irritable when restricted from going out					
	Always conform	1.208	149.631	3.348(2.759–4.063)	<0.001
	Often conform	0.937	115.492	2.553(2.152–3.028)	<0.001
	Sometimes conform	0.648	122.920	1.911(1.704–2.143)	<0.001
	Do not conform	0.000		1	
The time spent on electronic devices for entertainment was prolonged					
	Always conform	1.914	346.763	6.779(5.542–8.291)	<0.001
	Often conform	1.348	299.414	3.851(3.306–4.487)	<0.001
	Sometimes conform	0.727	139.908	2.090(1.849–2.361)	<0.001
	Do not conform	0.000		1	
Residence					
	FuZhou	0.345	5.207	1.412(1.050–1.899)	0.022
	XiaMen	0.293	12.676	1.340(1.141–1.574)	<0.001
	QuanZhou	0.348	21.086	1.416(1.220–1.642)	<0.001
	LongYan	0.311	5.265	1.365(1.046–1.780)	0.022
	NanPing	0.181	1.112	1.199(0.856–1.679)	0.292
	NingDe	0.19	0.383	1.210(0.662–2.211)	0.536
	PuTian	0.258	6.902	1.295(1.068–1.570)	0.009
	SanMing	0.791	18.399	2.207(1.537–3.168)	<0.001
	ZhangZhou	0.000		1	

^a^*β* is the regression coefficient of logistic regression analysis, which indicates how many units a dependent variable increases or decreases if the other independent variables are unchanged. ^b^Wald *χ^2^* is equal to *β* divided by its standard error squared, and is used to test whether the partial regression coefficient *β* of logistic whether is equal to 0 (The rows with *β* of 0 and OR of 1 in Table 5 was used as controls.). OR, Odds ratio; CI, Confidence interval.

**Figure 5:**
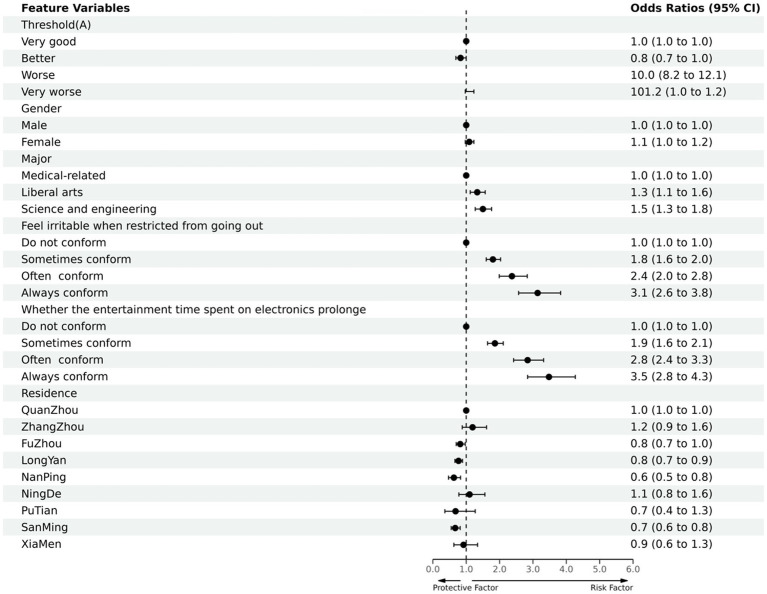
Protective and risk factors of multi-factor logistic regression analysis of sleep quality among college students (Sleep quality/A).

The results of the analysis (Table 4 and Figure 5) showed that the sleep quality of females was worse than males during the COVID-19 epidemic; The sleep quality of the population of liberal arts and science and engineering was worse than medical-related, especially in the liberal arts; The sleep quality of those who felt irritable with restricted access to the outside and those with prolonged electronic entertainment time were both worse than the average population. The variables follow a hierarchical order of sometimes conforming, often conforming, and always conforming. There was no statistically significant difference in sleep quality between the study districts.

**Figure 6:**
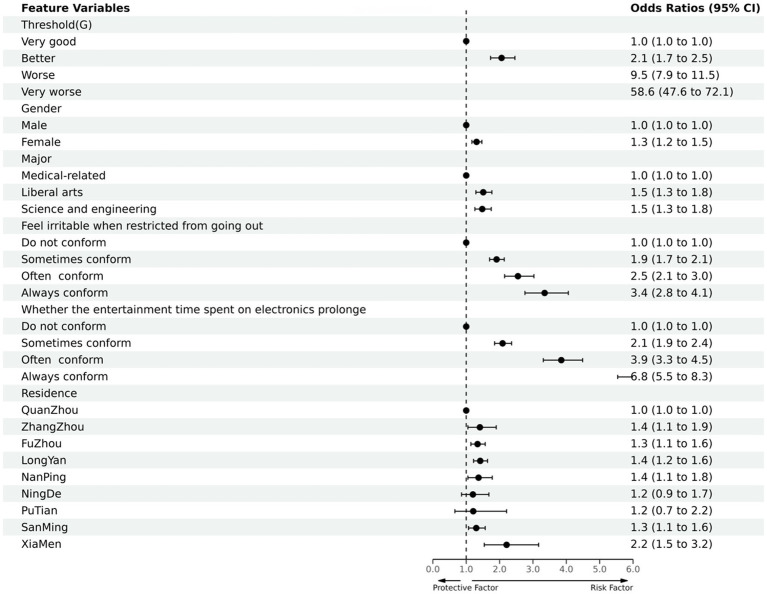
Protective and risk factors of multi-factor logistic regression analysis of sleep quality among college students (Daytime dysfunction/G).

The other results (Table 5 and Figure 6) showed that the proportion and degree of daytime dysfunction in men were lower than those in women; Daytime dysfunction in medical-related majors was better than that in liberal arts and science and engineering, with liberal arts being more serious than science and engineering; Daytime dysfunction in those who felt irritable when they were restricted from going out and those who spent more time on electronic entertainment showed different degrees of increase compared with the normal population, and the variables that are sometimes conforming, often conforming, and always conforming are progressive step-by-step; The daytime dysfunction of the population in Fuzhou city, Xiamen city, and Longyan city were more severe than that of the normal population, while the population in other district had no statistical significance.

## 4. Discussion

This is the first study to explore the relationship between sleep quality and daytime dysfunction in Chinese people during the COVID-19 epidemic. We found that Sleep quality and daytime dysfunction among Chinese college students were affected during the COVID-19 epidemic and were more severe than before the outbreak of COVID-19. We found that the average PSQI score of the tested Chinese college students was 6.17 ± 3.263, the sleep disorder rate was 29.6% (PSQI score>7), the daytime dysfunction rate was 85% (Daytime dysfunction score ≥ 1. Among them The percentage of daytime dysfunction scores 1–2 was 31%, scores 3–4 was 33%, scores 5–6 was 21%). In terms of demographic characteristics, the sleep quality of the group with female, liberal arts and science and engineering was worse than that of other groups, and the occurrence of daytime dysfunction was higher than other groups. Logistics regression analysis showed that sleep quality and daytime dysfunction were associated with gender, profession, irritable (due to limited outdoor), and prolonged electronic entertainment time.

Due to the rapid development of science and engineering, social life is changing rapidly, and the social pressure faced by each person has increased steeply (4, 13). Sleep problems and daytime dysfunction can often appear under these pressures (Among the daytime dysfunction are the following (14): ①Weakness or overall illness; ②Diminished ability to pay attention, maintain focus, or remember things; ③Diminished capacity for learning, working, or socializing; ④Erratic or irritable moods; ⑤Daytime drowsiness; ⑥Loss of Interest and Energy; ⑦Increased propensity to make mistakes while working or driving; ⑧Tension, headache, or other somatic symptoms associated with sleep loss; ⑨Excessive worry about sleep.) (6, 12, 15). The number of college students group continues to increase. Employment pressure, academic pressure, interpersonal pressure, and other problems continue to emerge under the influence of the COVID-19 epidemic. In such cases, the sleep problems of this group must be addressed and taken seriously (16). A survey by scholars (6, 17) in 2018 showed that the prevalence of sleep disorders was 18.3%, a survey in 2019 showed that the prevalence of sleep disorders was 33.3%, and a survey in 2020 showed that the prevalence of sleep disorders was 35.1%. The “2021 China Sleep Index Report” released by China in 2021 also showed that sleep problems are becoming more prevalent and severe. Since the COVID-19 epidemic, the sleep quality of college students is gradually decreasing, the rate of sleep disorders continues to increase, and daytime dysfunction is growing.

In the context of the recurrence of the COVID-19 epidemic, this study found that the population of those with very good and better sleep quality in component A of the PSQI scale accounted for 40%, and its proportion was not much different from the proportion of those with very good sleep quality corresponding to the total PSQI score, indicating that the PSQI evaluation has a certain degree of reliability and authenticity (9). In the component BCD, the cumulative score of sleep time was 86% for people with less than 4 points; The sleep time was 84% for people with more than 6 h of sleep, but only 32% of them had more than 7 h; The sleep efficiency was 82% for people with more than 75%, but only 52% of them had more than 85% efficiency. The difference between the three ratios is not significant, which suggests that because patients have longer sleep time and ordinary sleep efficiency, then the sleep time also decreases to a different extent. This may be due to the fact that during the COVID-19 epidemic period, some college students had large mood swings and failed to regulate their emotions in a timely and reasonable manner, and they passively coped with the changes brought about by the COVID-19 epidemic, which affected sleep time and sleep efficiency in a certain extent, and thus reduced sleep time when the next day’s study and living time was scheduled (5, 18). In the accompanying component EG, only 14% of the population had a cumulative score of 0 for a sleep disorder, and the remaining 86% of the population had different degrees of sleep disorder, of which 53% of the population had a cumulative score of 10 or more; At the same time, only 15% of the population had a cumulative score of 0 for daytime dysfunction,85% of the population had different degrees of daytime dysfunction, and 54% of the population had more serious dysfunction. The percentage of people with different degrees of daytime dysfunction was 85%, and the percentage of people with more serious dysfunction was 54%. This suggests that shortened sleep duration and reduced sleep efficiency can lead to different degrees of sleep disorders and daytime dysfunction compared to before the COVID-19 epidemic (1–3, 19, 20). On the one hand, this may be due to the fact that most college students are unable to arrange their studies and lifetime rationally in the context of the recurrent COVID-19 epidemic and failed to actively cope with the adverse effects of the COVID-19 epidemic (21). Thus, on the basis of the shortened sleep time and reduced sleep efficiency, they are unable to regulate their own life rhythm which resulted in the successive emergence of sleep disorders and daytime dysfunction; On the other hand, it either stems from the fear of uncertainty and variability of the COVID-19 epidemic, combined with the failure to relieve it in a timely manner, which in turn causes increased sleep disturbances and daytime dysfunction (22, 23).

It is important to note that the number of people using hypnotic drugs in Component F was only 5%, which indicates that even though the surveyed population had a significant degree of poor sleep quality, they were still reluctant to use hypnotic drugs (such as Estazolam, Zopiclone, Oryzanol Tablets, and so on). But perhaps it was the reluctance to use drugs that led to the worsening of sleep problems and coupled with the complex environmental impact of the epidemic, which in turn led to a continued decrease in sleep quality (5, 24, 25). On the basis of the total PSQI score, the comparison of PSQI component A and G showed that the proportion of people with very good or better sleep quality and those with no or little daytime dysfunction was similar, which means to some extent that people with good sleep quality tend to be energetic in their daily work and study life and rarely have daytime dysfunction such as sleepiness. This is probably because these people can correctly cope with the adverse impact of the COVID-19 epidemic, timely grasp their own life and learning rhythm, and actively overcome the challenges brought by changes in the surrounding environment to ensure that they are only minimally affected or even not affected by the COVID-19 epidemic in the context of recurrent epidemics (2, 5, 6, 26, 27).

The results of this study showed that women had higher rates of sleep disturbance and poorer sleep quality than men during the COVID-19 epidemic and had a higher proportion and severity of daytime dysfunction than men, which may be due to the physiological characteristic that women are more sensitive to the external environment and have higher risk perception than men, and this characteristic may be exacerbated in response to environmental changes in the context of recurrent COVID-19 epidemics (5, 28). It is possible that the medical-related population has better sleep quality and less daytime dysfunction than the liberal arts and science and engineering, possibly because the medical-related population has a more comprehensive and specific understanding of the COVID-19 epidemic and can make better judgments and more appropriate adjustments (29). Among them, the poor sleep quality and daytime dysfunction of the liberal arts population are more severe than those of the science and engineering population, probably because the liberal arts population perceives changes more carefully and thus responds to environmental changes in a more complex way, while the science and engineering population is less affected because of a deeper understanding of the inner nature of things (4, 5, 28, 29). Those who feel irritable when they are restricted from going out and those who spend more time on electronic entertainment have poorer sleep quality than other groups, and daytime dysfunction is also more severe than other groups. This is probably due to the fact that the quality of sleep was reduced because of the restriction of going out, irritability, and the reduction of recreational activities (5, 6); Based on this situation, the use of electronic devices is prolonged to relax, but the next day’s class time is relatively fixed, thus affecting the quality of sleep, and in a vicious circle, which in turn affects daytime dysfunction (4–6, 29). It is worth mentioning that this phenomenon is more significant in the district where the COVID-19 epidemic is recurrent, such as Fuzhou city and Xiamen city. In these cities, the COVID-19 epidemic is being prevented and controlled with stronger procedures, such as prohibiting students from leaving school except in life-threatening situations and quarantining and monitoring visitors and departing campus for at least 2 weeks (Before the COVID-19 outbreak, these procedures had never been implemented).

It should be noted that the proportion of those who always and often conformed to the restriction of going out and feeling irritable and the prolongation of electronic entertainment increased significantly compared to those who did not, and the proportion of poor sleep quality and daytime dysfunction that accompanied them also increased significantly, while gender and major had a certain degree of influence on sleep quality and daytime dysfunction, but less than the two. This suggests that it is the main reason for the emergence of poor sleep quality and daytime dysfunction among college students during the COVID-19 epidemic. This may be because the COVID-19 epidemic had a negative impact on the cognitive ability of some college students, which led to their irritability and depression, and the superimposed effect of psychological factors such as irritability and prolonged electronic entertainment time, in which sleep disorders and daytime dysfunction emerged and worsened one after another (19, 21, 26, 29); On the contrary, although factors such as gender and major all had an impact to some extent, the superimposed possibility or superimposed effect due to the epidemic was relatively small (19, 29).

In conclusion, the situation of sleep quality and daytime dysfunction is not optimistic among college students in the context of recurrent epidemics. It is urgent to implement individualized measures to alleviate and improve the physical and mental health of college students, such as correctly guiding college students’ cognition of the COVID-19 epidemic, establishing confidence in overcoming the COVID-19 epidemic, providing social support to relieve the stress load, carrying out special lectures and publicity, holding offline fellowship activities on campus, applying a music therapy, and easing the epidemic control and prevention measures if circumstances permit in order to improve the current situation of poor sleep quality and daytime dysfunction among college students caused by the COVID-19 epidemic (1, 10, 17, 30).

It is noteworthy to mention that colleges and universities worldwide implemented diverse precautionary measures at varying stages of the COVID-19 situation. A number of colleges and universities around the world adopted stringent COVID-19 prevention measures such as mandatory mask-wearing on campus, promoting distance learning, and suspending offline experiments (31–33). While students’ sociocultural backgrounds and customs might differ, it was observed that many experienced sleep disturbances due to concerns regarding the epidemic, familial issues, and academic performance (3–6, 32, 33).

## 5. Strengths and limitations

This study’s strength was its substantial sample size, which gave it the power to find significant relationships. A questionnaire is also more practical and economical for a large-scale population survey, although having a potential for lower specificity than laboratory tests.

Some potential limitations of the study merit discussion. First, the causality of the observed relationships in this study could not be established because of the cross-sectional nature of the study. Second, all sleep quality and daytime dysfunction information were collected via self-reporting, and objective measures of sleep quality and daytime dysfunction were not available in the study. Misclassification and recall bias could be introduced. Third, the scope of the clinical implications of our findings were constrained by the subjective global evaluation of sleep quality and daytime dysfunction in this investigation. Fourth, worse sleep quality and daytime dysfunction may be related not only to home isolation and offline learning due to the epidemic, but it is also possible that it is related to poor lifestyle habits of the subject population, such as smoking, drinking, staying up late, and irregular work and rest (34, 35). In addition, it should be noted that the COVID-19 epidemic also exacerbates these poor habits. Since women are more susceptible to environmental influences than men, and the recurrence of epidemics brings about enormous environmental changes, the large and substantial depression in sleep quality does not exclude the reason for the predominance of women in the subject population (36).

## 6. Conclusion

During the COVID-19 epidemic, college students’ sleep quality has been affected, with varying degrees of daytime dysfunction, both are in worse condition than before the COVID-19 outbreak. Sleep quality may have been negatively associated with daytime dysfunction.

## Data availability statement

The original contributions presented in the study are included in the article/supplementary material, further inquiries can be directed to the corresponding author.

## Ethics statement

The studies involving humans were approved by the Second Affiliated Hospital of Fujian Medical University. The studies were conducted in accordance with the local legislation and institutional requirements. The participants provided their written informed consent to participate in this study.

## Author contributions

WJ: Data curation, Formal analysis, Investigation, Methodology, Software, Validation, Writing – original draft. LS: Funding acquisition, Investigation, Methodology, Project administration, Supervision, Validation, Writing – original draft. XL: Formal analysis, Investigation, Writing – original draft. ZS: Investigation, Writing – original draft. QC: Formal analysis, Writing – original draft. DS: Methodology, Writing – original draft. PH: Formal analysis, Investigation, Writing – original draft. ZZ: Data curation, Investigation, Writing – original draft. JF: Investigation, Writing – original draft. YH: Investigation, Writing – original draft. MX: Investigation, Writing – original draft. JY: Methodology, Writing – original draft. XC: Formal analysis, Funding acquisition, Project administration, Supervision, Validation, Writing – review & editing.
